# Supplementary material for: Dissection of intercellular communication using the transcriptome-based framework ICELLNET
Source: Nat Commun. 2021 Feb 17;12:1089. doi: 10.1038/s41467-021-21244-x (PMC7889941; doi:10.1038/s41467-021-21244-x)
Supplement: Supplementary file 1 — Supplementary Information [file 41467_2021_21244_MOESM1_ESM.pdf]

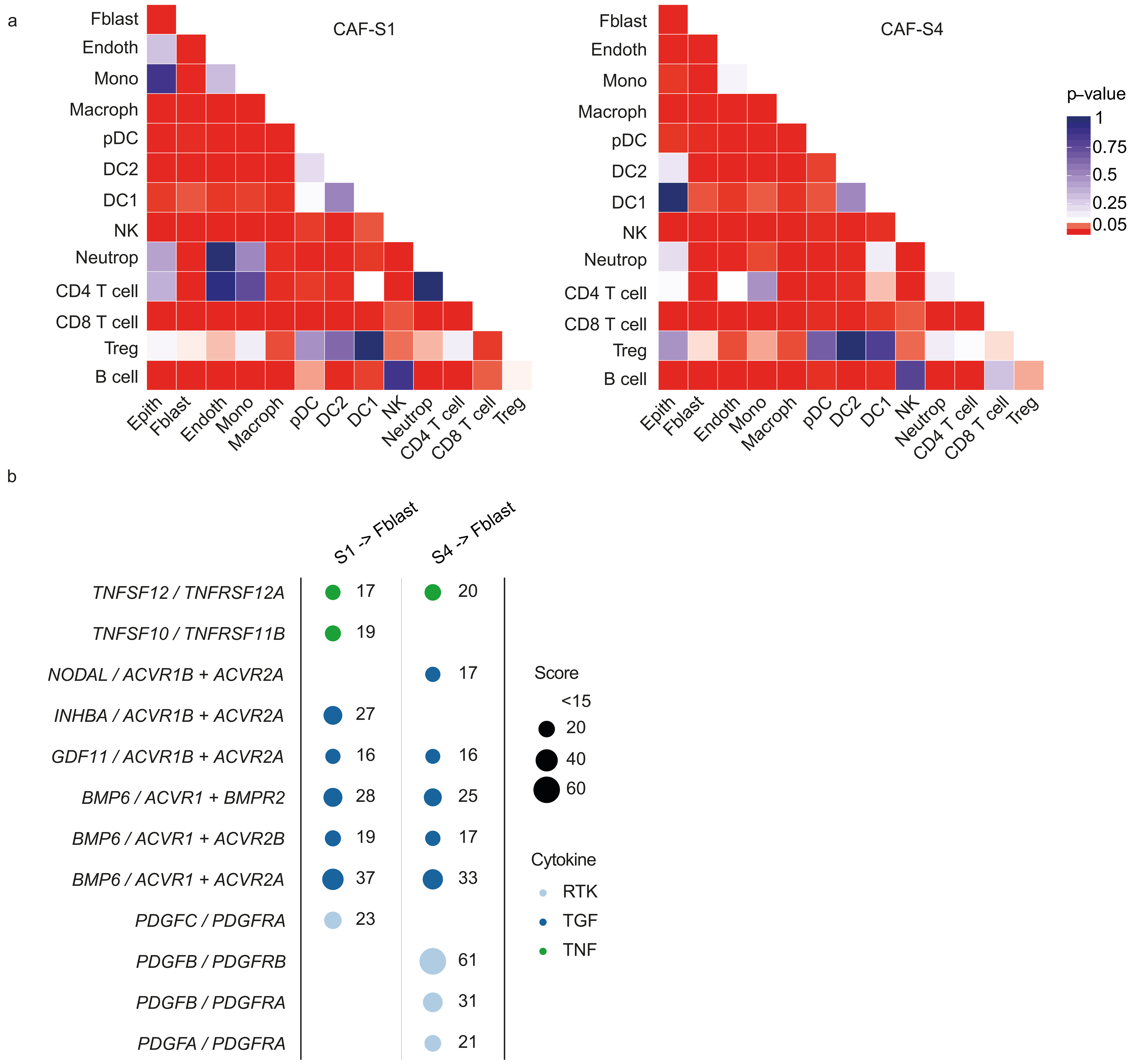

Supplementary Figure 1: Comparison of cytokine-mediated intercellular communication between Triple-Negative breast cancer infiltrating CAF subsets CAF-S1 and CAF-S4. (a) Statistical score comparison (two-sided wilcoxon test, and pvalues are adjusted with Benjamin-Hochberg method) of the outward cytokine-mediated communication scores computed from CAF-S1 (n=6 biologically independent samples) to the different partner cells (left), and the outgoing communication scores from CAF-S4 (n=3 biologically independent samples) to the different partner cell types (right). (b) Individual outward communication score of specific ligand-receptor interactions that contribute to communication scores from CAF-subsets to breast fibroblasts with a score superior or equal to 15.

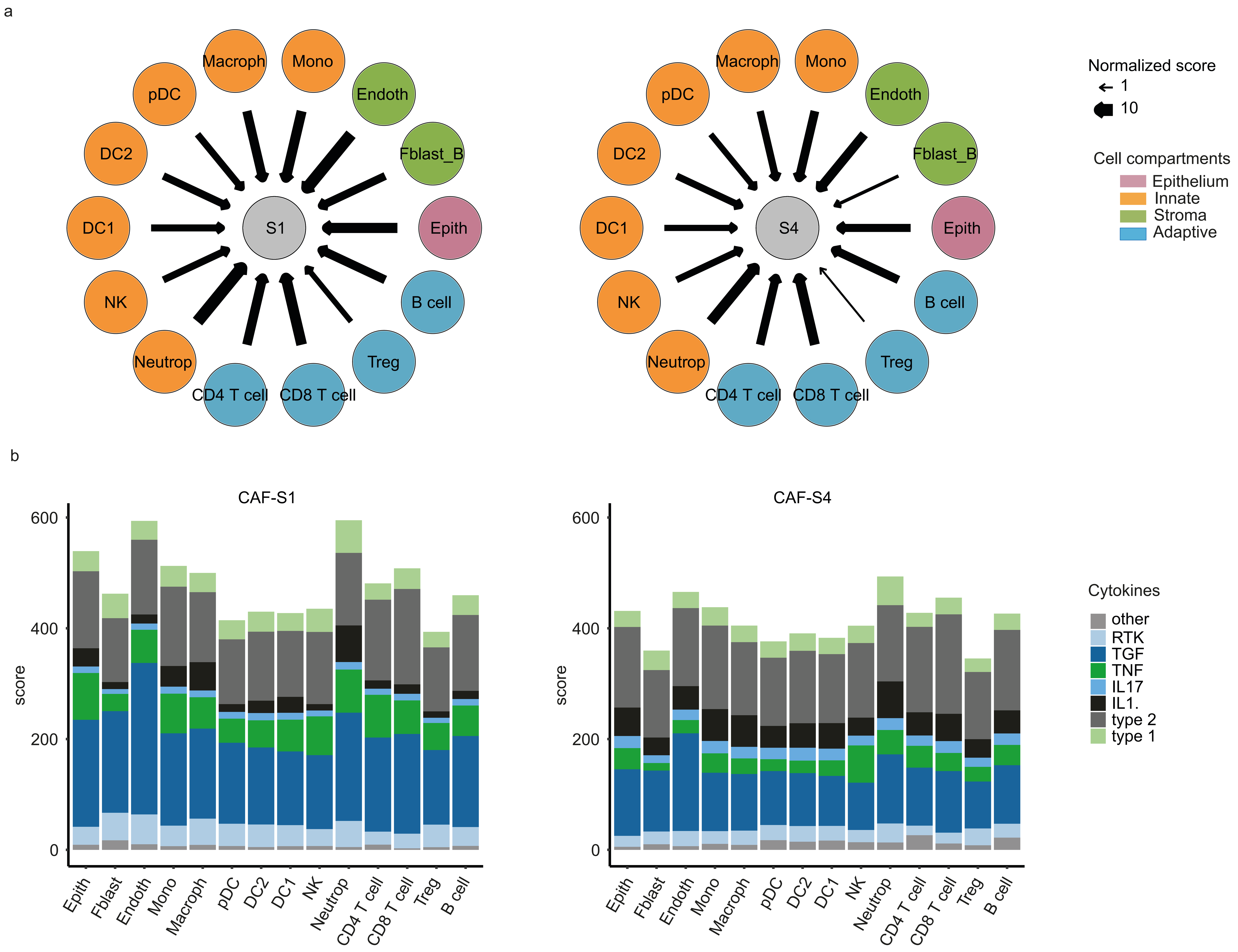

Supplementary Figure 2: Dissecting inward communication of Triple Negative CAF-subsets from putative cell types present in the tumor microenvironment (a) Connectivity maps describing inward cytokine-mediated communication from primary cells to CAF-S1 (n=6 biologically independent samples) and CAF-S4 (n=3 biologically independent samples) subsets. The CAF subsets are considered as central cells and colored in grey. Primary cells are considered as partner cells and are colored depending on the cell compartment (green: stroma, orange: innate, blue: adaptive, pink: epithelium). The width of the edges corresponds to a global score combining the intensity of all the individual ligand-receptor interactions. A scale ranging from 1 to 10, corresponding to minimum and maximum communication scores, is shown in the legend. (b) Barplot of communication score with contribution restricted to cytokines subfamilies between CAF subsets and a selection of partner cells (CAF-S1 n=6 biologically independent samples, CAF-S4 n=3 biologically independent samples).

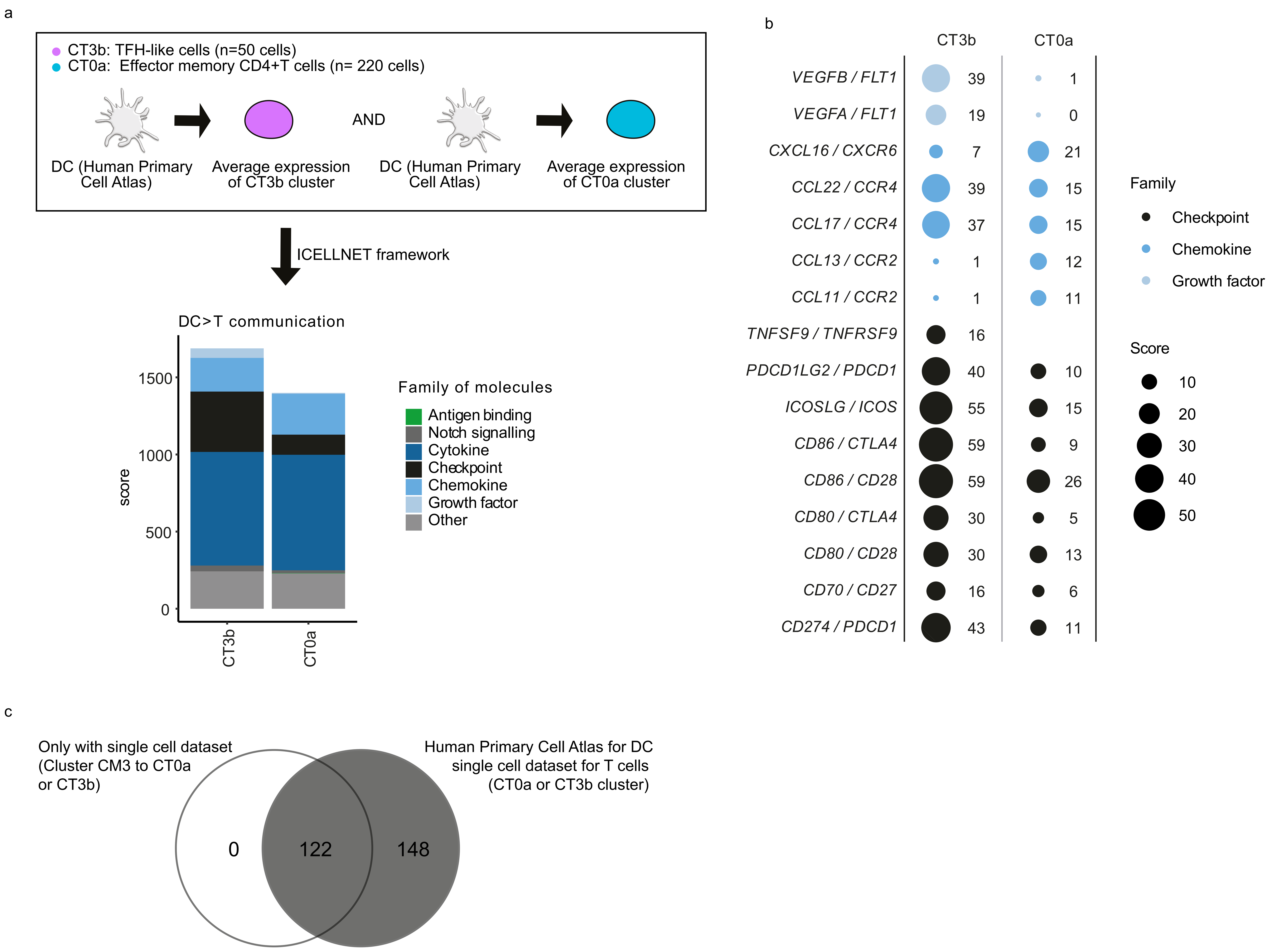

Supplementary Figure 3: Study of cell-to-cell communication potential between dendritic cells and T cell subpopulations in lupus nephritis single-cell data using Human Primary Cell Atlas profiles for conventional dendritic cells. (a) ICELLNET framework applied to assess DC - T intercellular communication, between two T cells clusters from the single-cell datasets (CT3b and CT0a), and dendritic cells profiles from Human Primary Cell Atlas (n= 70 biologically independent samples over 7 independent experiments). For single-cell data, average expression profiles were computed from the single-cell data matrix counts for each cluster. Barplots display the contribution of the different communication molecules families to the communication scores. (b) Ballon plot representing specific individual interaction scores that differs from at least 10 between the two conditions (cutoff chosen for clarity purpose) for interaction belonging to either chemokine, checkpoint or growth factor families of molecules. (c) Venn diagram displaying the number of ligand/receptor pairs contributing to the communication scores of lupus nephritis single-cell dataset towards cell populations coming from the same single-cell dataset (left) or the Human Primary Cell Atlas dataset (right). In the middle of the Venn are shown the number of common ligand-receptor pairs. Some schematic art pieces were used and modified from Servier Medical Art, licensed under a Creative Common Attribution 3.0 Generic License. http://smart.servier.com/ (a).

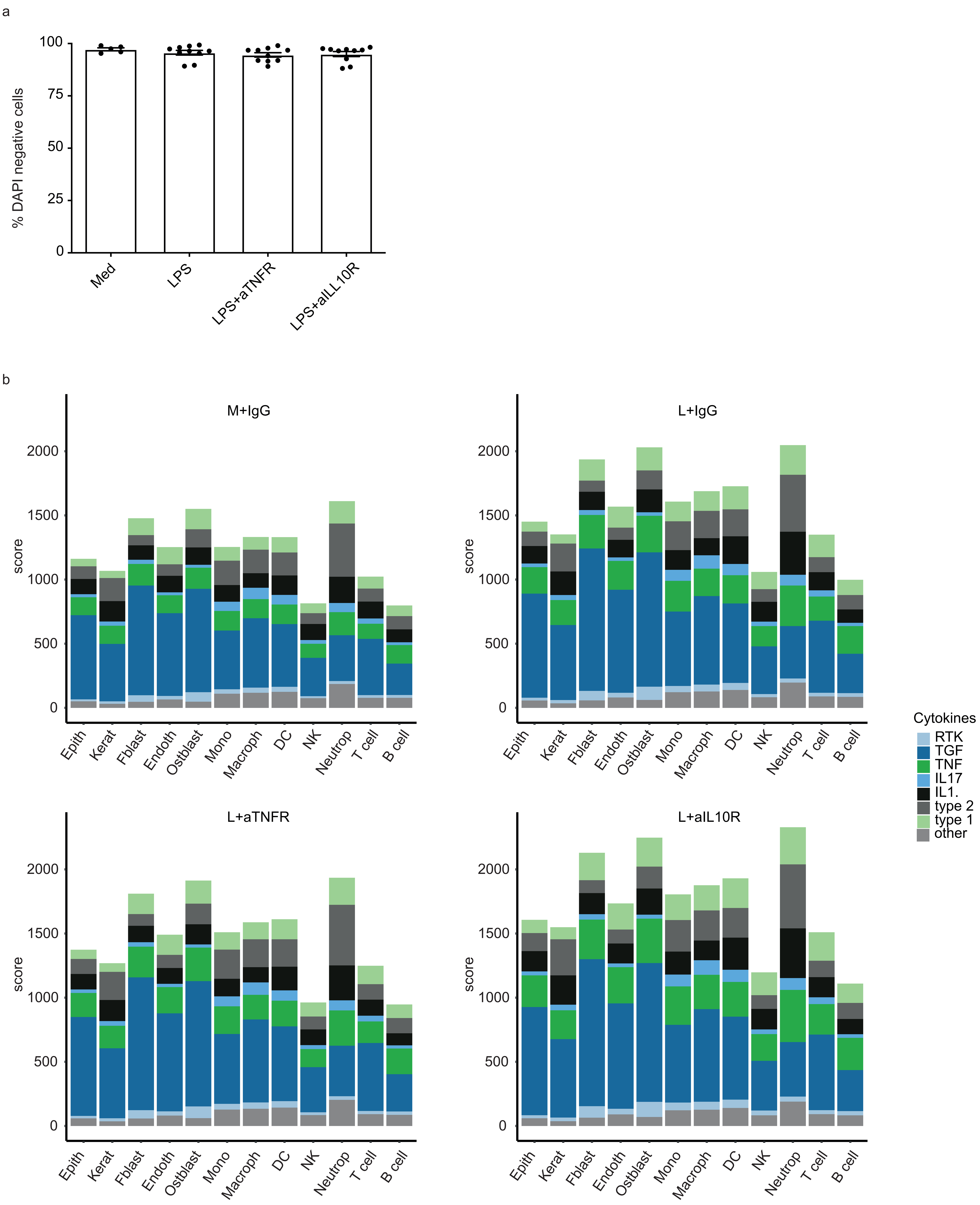

Supplementary Figure 4: IL-10R blocking activates a cell-to-cell communication module in LPS-stimulated DCs. (a) Cell viability of DC cultured 24 hours in the indicated blocking conditions (Medium, LPS activation, LPS and TNFR or IL-10R blocking antibodies) was assessed by DAPI staining (n=6 biologically independent samples). Data represented are mean values ± SEM of DAPI negative cell percentage. (b) Barplot of communication score with contribution by families of communication molecules between in vitro activated DCs in each biological condition and a selection of partner cells (n= 6 biologically independent samples).

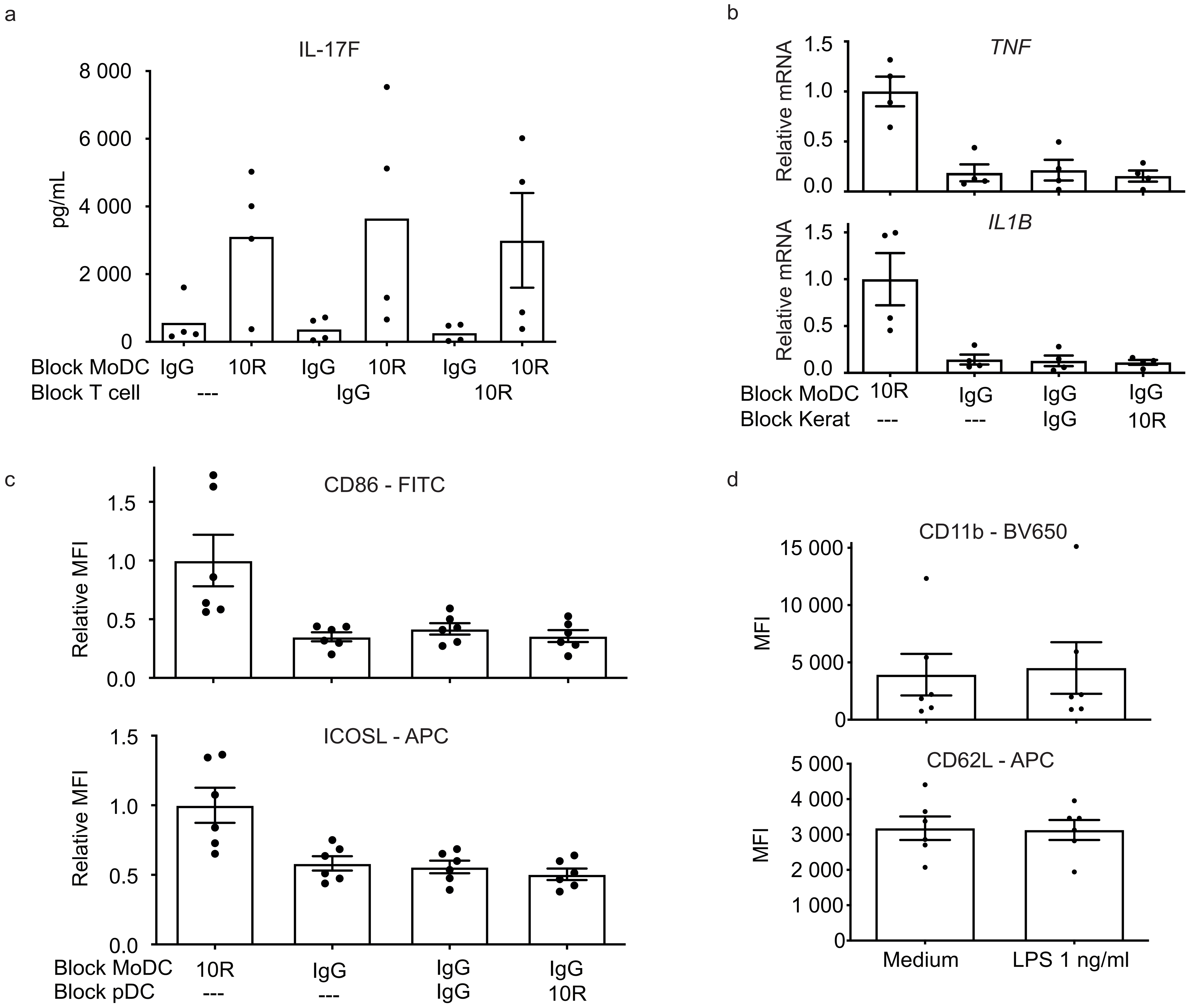

Supplementary Figure 5: Observed effect on communication partner-cells is not due to the presence of residual aIL-10R antibody or a potent LPS dose. (a) CD4 Naive T cells were pre- treated with blocking antibody for IL-10 receptor or a non-specific one and then put in culture with DC as indicated for 6 days. After restimulation with anti-CD3/anti-CD28 for 24 hours, supernatants were analyzed for the presence of IL-17F. Data are represented as mean values +/- SEM (n= 4 biologically independent samples). (b) HaCat cells were pre-treated with blocking antibody for IL-10 receptor or a non-specific one and then put in culture with DCs supernatant (diluted 1:10) as indicated for 4 hours. RNA was then extracted from cells and the expression of *TNF* and *IL1B* was assayed using qRT-PCR (n=4 biologically independent samples) Data are represented as mean values +/- SEM. (c) pDCs were pre-treated with blocking antibody for IL-10 receptor or a non-specific one and then put in culture with DCs supernatant (diluted 1:10) as indicated for 24h. Expression of maturation markers CD86 and ICOSL analyzed by flow cytometry (n= 6 biologically independent samples). Data are represented as mean values +/- SEM. (d) Neutrophils cultured for 1h with 1 ng/ml LPS were not significantly activated compared to medium as assessed by surface expression of CD11b and CD62L by flow cytometry (n=6 biologically independent samples). Data are represented as mean values +/- SEM.

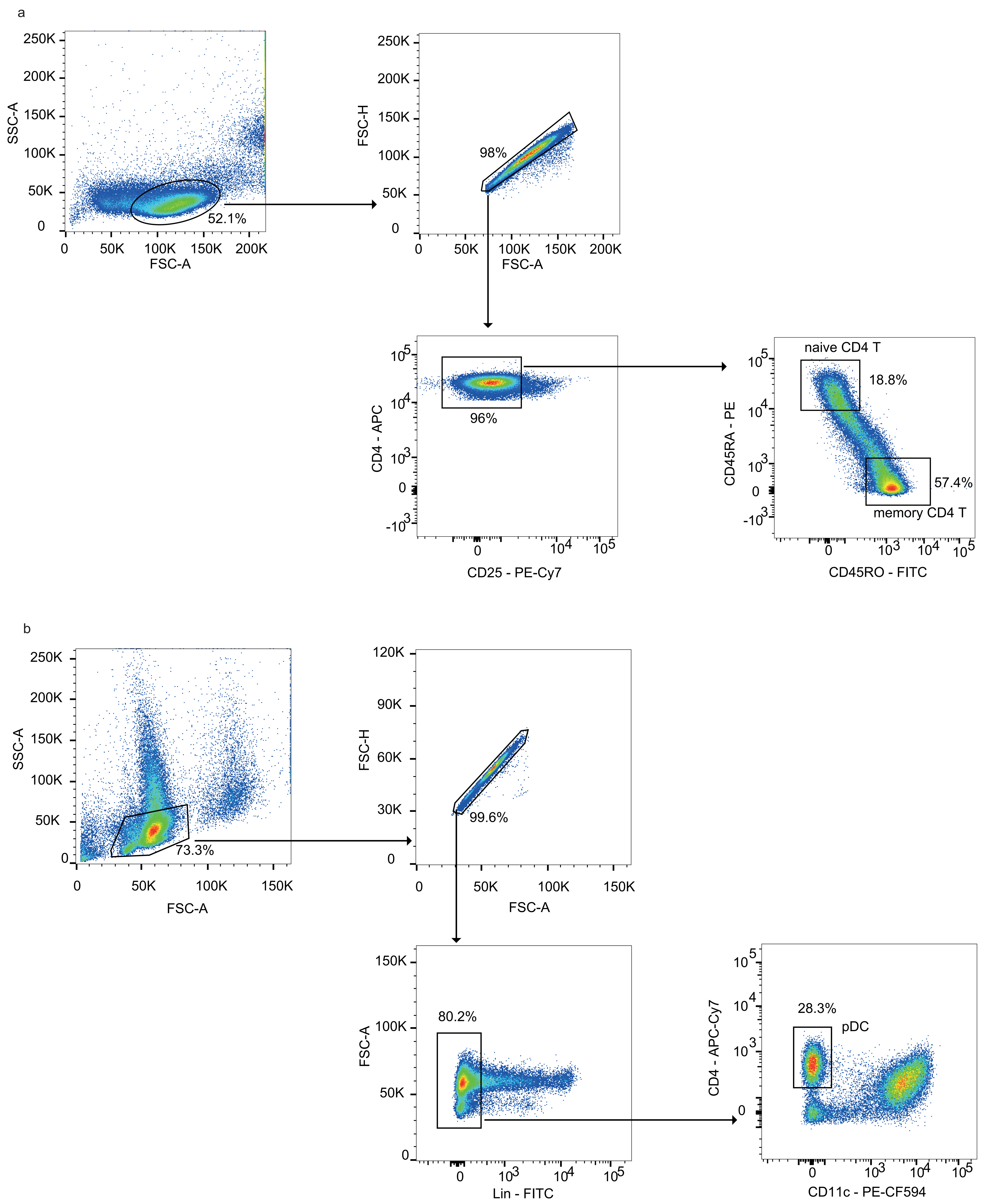

Supplementary Figure 6: Representative gating strategy used in the current study for sorting of specific peripheral blood mononuclear cell types from PBMCs fractions. (a) Gating strategy used for naïve/memory CD4 T cells sorting from CD4 T cells. Cells were gated to remove debris and doublets. Among the CD25-CD4+ cells, we selected CD45RA+CD45RO- cells as naive CD4+ T cells, and CD45RA-CD45RO+ cells as memory CD4+ T cells. (b) Gating strategy used for plasmacytoid dendritic cells from DC-enriched PBMCs (magnetic enrichment). Cells were gated to remove debris and doublets. Plasmacytoid dendritic cells were gated as Lin-CD4+CD11c- (Lin=CD4, CD14, CD16, CD19)

**Gene**	**Ref_seq**
ACTB	NM_001101
GAPDH	NM_002046.3
RLP34	NM_000194
TNF	NM_000594
IL1B	NM_000576

Supplementary Table 1: List of all TaqMan probes (Life Technologies) used for RT-qPCR
