# Supplementary material for: Dissection of intercellular communication using the transcriptome-based framework ICELLNET
Source: Nat Commun. 2021 Feb 17;12:1089. doi: 10.1038/s41467-021-21244-x (PMC7889941; doi:10.1038/s41467-021-21244-x)
Supplement: Supplementary file 3 — Description of Additional Supplementary Files [file 41467_2021_21244_MOESM3_ESM.pdf]

Description of Supplementary Data

Supplementary Data 1: Manually curated database of ICELLNET ligand-receptor interactions. The database is structured as following: column 1-5, gene symbol for ligand/receptor subunits, column 6, possible aliases for ligands and receptors, column 7-9, classification into families of molecules, column 10, PubMed ID corresponding to the interaction.

Supplementary Data 2: Description of the Human Primary Cell Atlas dataset integrated in ICELLNET package. It contains a total 745 transcriptomic profiles among 31 cells types generated with the same Affymetrix technology.

Supplementary Data 3: (a) CAF subsets outward communication scores considering all the ligand-receptor database, including raw global communication score, global communication score after rescaling, communication score of each family of molecules, and statistical analysis. (b) CAF outward individual communication scores with selected partner cells. (c) CAF subsets outward cytokine-mediated communication scores, including raw global communication score, global communication score after rescaling, communication score of each subfamily of cytokines, and statistical analysis (two-sided wilcoxon test, and pvalues are adjusted with Benjamin-Hochberg method).

Supplementary Data 4: (a) Global outward communication score from CM3 cluster (cluster average expression) to CT0a and CT3b clusters (cluster average expression) and contribution of each family of molecules to the communication scores. (b) Outward individual communication scores between CM3 cluster and CT0a and CT3b clusters. (c) (left) Communication scores (CM3 to CT3b and to CT0a) and standard deviation for CM3 cluster subsampling (n=20 times), (right) Communication scores (CM3 to CT3b and to CT0a) and standard deviation for CT3b and CT0a cluster subsampling (n=20 times).

Supplementary Data 5: (a) Global outward communication score from the in vitro-activated DCs to selected partner cells, including raw global scores, and global scores after rescaling, steps. (b-e) List of individual outward communication scores from each DCs biological condition to selected partner cells. (f) Differential expression analysis was performed using an ANOVA test (Matlab function anova1) for these two time points. P-values were adjusted for

multiple testing using the Benjamini-Hochberg correction using the Matlab function mafdr. Adjusted p-values <5% were considered significant.

Supplementary Data 6: Expression of maturation markers CD86, HLA-DR and ICOSL or CD11b and CD62L analyzed by flow cytometry with surface staining on (a) pDCs (n=18 or n=6 biologically independent samples depending on the conditions), (b) keratinocytes (n=8 or n=4 biologically independent samples depending on the conditions), and (c) neutrophils (n=9 or n=6 biologically independent samples depending on the conditions) cultured with supernatant of the indicated DC. (d) Statistical tests results (two-sided paired t-test, pvalues are adjusted with Benjamin-Hochberg method).
