# Supplementary material for: Dissection of intercellular communication using the transcriptome-based framework ICELLNET
Source: Nat Commun. 2021 Feb 17;12:1089. doi: 10.1038/s41467-021-21244-x (PMC7889941; doi:10.1038/s41467-021-21244-x)
Supplement: Supplementary file 10 — Reporting Summary [file 41467_2021_21244_MOESM10_ESM.pdf]

nature research

Corresponding author(s): Vassili Soumelis

Last updated by author(s): Dec 13, 2020

Reporting Summary

Nature Research wishes to improve the reproducibility of the work that we publish. This form provides structure for consistency and transparency in reporting. For further information on Nature Research policies, see Authors & Referees and the Editorial Policy Checklist.

Statistics

For all statistical analyses, confirm that the following items are present in the figure legend, table legend, main text, or Methods section.

n/a	Confirmed	
☐	☒	The exact sample size (*n*) for each experimental group/condition, given as a discrete number and unit of measurement
☐	☒	A statement on whether measurements were taken from distinct samples or whether the same sample was measured repeatedly
☐	☒	The statistical test(s) used AND whether they are one- or two-sided<br>*Only common tests should be described solely by name; describe more complex techniques in the Methods section.*
☒	☐	A description of all covariates tested
☐	☒	A description of any assumptions or corrections, such as tests of normality and adjustment for multiple comparisons
☐	☒	A full description of the statistical parameters including central tendency (e.g. means) or other basic estimates (e.g. regression coefficient) AND variation (e.g. standard deviation) or associated estimates of uncertainty (e.g. confidence intervals)
☐	☒	For null hypothesis testing, the test statistic (e.g. *F*, *t*, *r*) with confidence intervals, effect sizes, degrees of freedom and *P* value noted<br>*Give P values as exact values whenever suitable.*
☒	☐	For Bayesian analysis, information on the choice of priors and Markov chain Monte Carlo settings
☒	☐	For hierarchical and complex designs, identification of the appropriate level for tests and full reporting of outcomes
☒	☐	Estimates of effect sizes (e.g. Cohen's *d*, Pearson's *r*), indicating how they were calculated

Our web collection on statistics for biologists contains articles on many of the points above.

Software and code

Policy information about availability of computer code

Data collection	For the Human Primary Cell Atlas dataset, we downloaded the already processed and normalized dataset.<br><br>Cells corresponding to CAF-S1 and CAF-S4 have been isolated, collected, and sequenced. Reads were mapped on the reference genome (hg19/GRCh37 from UCSC genome release) using Tophat_2.0.6 algorithm. RNAseq CAF dataset is publicly available.<br><br>Monocyte-derived DCs were pre-treated with blocking Abs as described above for one hour, cultured with medium or LPS for an additional 4 or 8 hours. Total RNA was extracted using the RNeasy micro kit (Qiagen). Samples were then amplified and labelled according to the protocol recommended by Affymetrix for hybridization to Human Genome U133 Plus 2.0 arrays.
Data analysis	Most analysis were performed on R (version 3.1 and 3.6.3). RNAseq gene expression quantification was performed using HTSeq-Count and featuresCount. Only genes with five reads in at least 25% of all samples were kept for further analyses. Normalization was done using the method implemented in DESeq2 package (version 1.26.0).<br>For Affymetrix data, the gene expression data were normalized with Plier and further transcriptomic analyses were performed in Matlab (version R2010).<br><br>All packages used on R for analyses : rlist (version 0.4.6.1), scales (version 1.1.0), ggthemes (version 4.2.0), gridExtra (version 2.3), , RColorBrewer (version 1.1-2), gplots (version 3.0.3), GGally (version 1.5.0), ggplot2 (version 3.3.0), psych (version 1.9.12.31), , jetset (version 3.4.0), annotate (version 1.64.0), hgu133plus2.db (version 3.2.3), org.Hs.eg.db (version 3.10.0) AnnotationDbi (version 1.48.0), BiocGenerics, (version 0.32.0), icellnet (version 0.0.0.9000), DESeq2 (version 1.26.0), stats (version 3.6.1).<br><br>Code corresponding to some analyses and functions developed for ICELLNET package is available on Github at https://github.com/soumelis_lab/ICELLNET

For manuscripts utilizing custom algorithms or software that are central to the research but not yet described in published literature, software must be made available to editors/reviewers. We strongly encourage code deposition in a community repository (e.g. GitHub). See the Nature Research guidelines for submitting code & software for further information.

Data

Policy information about availability of data

All manuscripts must include a data availability statement. This statement should provide the following information, where applicable:

- Accession codes, unique identifiers, or web links for publicly available datasets
- A list of figures that have associated raw data
- A description of any restrictions on data availability

The gene expression profiles generated for this publication have been deposited in NCBI's Gene Expression Omnibus and are accessible through GEO Series accession number GSE89342 (http://www.ncbi.nlm.nih.gov/geo/query/acc.cgi?acc=GSE89342).
The CAFs dataset has been published by Costa et al. 2018, and is accessible through the accession number EGAS00001002508 (https://www.ebi.ac.uk/ega/studies/EGAS00001002508) .
The single cell dataset of immune cells from lupus nephritis patients has been published by Arazi et al.2019, and is accessible through the ImmPort repository (accession code SDY997, https://www.immport.org/shared/study/SDY997).

In addition to the litterature, we manually curated the ligand-receptor database using STRING (http://string-db.org/), Ingenuity (http://www.ingenuity.com/), BioGRID (https://thebiogrid.org), Reactome (https://reactome.org) and CellPhoneDB databases (https://www.cellphonedb.org).

Field-specific reporting

Please select the one below that is the best fit for your research. If you are not sure, read the appropriate sections before making your selection.

☒ Life sciences ☐ Behavioural & social sciences ☐ Ecological, evolutionary & environmental sciences

For a reference copy of the document with all sections, see nature.com/documents/nr-reporting-summary-flat.pdf

Life sciences study design

All studies must disclose on these points even when the disclosure is negative.

Sample size	Sample size was determined based on availability and resource.
Data exclusions	No sample was excluded from the study.
Replication	We performed mainly biological replicates for the experiments (at least 3 biological replicates, often between 4 and 6, and up to 18 donors depending on the experiments). All attempts at replication were successful.
Randomization	All experiments were conducted from blood samples delivered by Crozatier Hospital (Etablissement Francais du Sang) without further information. All the patients were recruited randomly, and did not receive any treatments or intervention that could lead to assign them to groups.
Blinding	The blinding was not necessary in the current study, as the patients were randomly recruited, did not received any treatments and were not assigned into groups.

Reporting for specific materials, systems and methods

We require information from authors about some types of materials, experimental systems and methods used in many studies. Here, indicate whether each material, system or method listed is relevant to your study. If you are not sure if a list item applies to your research, read the appropriate section before selecting a response.

Materials & experimental systems

n/a	Involved in the study	
☐	☒	Antibodies
☐	☒	Eukaryotic cell lines
☒	☐	Palaeontology
☒	☐	Animals and other organisms
☐	☒	Human research participants
☐	☐	Clinical data

Methods

n/a	Involved in the study	
☒	☐	ChIP-seq
☐	☒	Flow cytometry
☒	☐	MRI-based neuroimaging

Antibodies

Antibodies used	For phenotypical characterization, single cell suspension was stained with the following anti-human antibodies: IFN gamma PE-Cy7 (eBioscience, cat# 25-7319-82, clone 4S.B3, lot E07674-1633) , IL-17A Alexa Fluor 488 (BioLegend, cat# 512308, clone BL168, lot B153596), IL-9 PerCP eFluor 710 (eBioscience, cat# 46-7098-41, clone MH9D1, lot E17114-101), CD86 FITC (BD, cat# 555657, clone 2331 (FUN-1), lot 19009), IgG1,κ FITC (BD, cat# 555748, clone 2331 (FUN-1), lot 2279908), HLA-DR Alexa Fluor 700 (Biolegend, cat# 307626, clone L243, lot B153402), IgG2a,κ Alexa Fluor 700 (Biolegend, cat# 400248, clone 400248, lot B153064), ICOSL APC (R&D Systems, cat# FAB165A, clone 136726), IgG2b,κ APC (R&D Systems, cat# IC0041A, clone 136726, lot LHD1314011), CD62L APC (BD, cat# 559772, clone 559772, lot 2276535), IgG1, κ APC (BD, cat# 555751, clone MOPC-21, lot 39573), CD15 PE (BD, cat# 555402, clone HI98), CD11b BV650 (Biolegend, cat# 101239, clone M1/70, lot B182602), Rat IgG2b, κ BV650 (Biolegend, cat# 400651, clone RTK4530, lot B172875)<br><br>For blocking experiments, anti-human blocking antibodies were provided by R&D Systems and the following anti-human blocking antibodies were used: Human TNF RI (MAB625, clone 16805, lot BHS1212041), Human TNF RII (MAB726, clone 22210, lot AYF0412081), Human IL-10 Rα (MAB274, clone 37607, lot ZL0612101), Goat IgG (AB-108-C, lot ES4113061 and ES4115041), Mouse IgG1 (MAB002, clone 11711, lot IX2413101 and IX214107), Mouse IgG2b (MAB004, clone 20116, lot NZ1015051), Human IL-19 (MAB10351, 152107, lot JER0208071), Human IL-36 γ (AF2320, lot UNN0112091), Human Oncostatin M (AB-295-NA, lot DZ0512011), Human TNF-α (MAB210, clone 1825, lot HM0709021), Human G-CSF (MAB214, clone 3316, lot GV1912031), Human GM-CSF (MAB215, clone 3209), Human IL-12p70 (MAB219, clone 24910, lot CIL081602), Human IL-6 (MAB206, clone 6708, lot HD32).<br><br>For pDC and CD4 memory and naive T cell sorting, the following anti-human antibodies were used: FITC Mouse anti-human CD3 (BD, Cat# 555339, clone HIT3a), FITC Mouse anti-human CD14 (Miltenyi Biotec, cat# 130-080-701, clone TÜK4), FITC Mouse anti-human CD16 (BD, cat# 335035, clone NKP15), FITC Mouse anti-human CD19 (Miltenyi Biotec, cat# 130-091-328, clone LT19), PE-Cy7 Mouse anti-human CD11c (Biolegend, cat# 337216, clone Bu15), PE-Cy5 Mouse anti-human CD4 (Beckman Coulter, cat# A07752, clone 13B8.2),<br><br>allophyco-cyanin-anti CD4 (VIT4; MiltenyiBiotec), phycoerythrin-anti-CD45RA (BD), fluorescein-isothiocyanate-anti-CD45RO (BD Bioscience) and phycoerythrin-7-anti-CD25 (BD bioscience)
Validation	All antibodies are established, well described and published elsewhere. Informations are avaible and accessible on the manufacturer's websites from the #cat number). Antibodies used were titred using peripheral blood mononuclear cells from healthy donors, or in DC-enriched fraction of PBMCs.

Eukaryotic cell lines

Policy information about cell lines

Cell line source(s)	We used the keratinocyte cell line HaCaT. This cell line was kindly provided by Prof. Dr. Bernhard Homey (Düsseldorf).
Authentication	None of the cell line used was not authenticated.
Mycoplasma contamination	All HaCaT cells were negative for Mycoplasma contamination, standardized and regular tests were performed by PCR for mycoplasma detection.
Commonly misidentified lines (See ICLAC register)	No commonly misidentified cell lines were used in the study.

Human research participants

Policy information about studies involving human research participants

Population characteristics	Blood from healthy human blood donors were obtained from Etablissement Francais du Sang (French Blood Establishment). Gender identity and age from anonymous donors were not available, but all donors were between 18 and 70 years old (age limits for blood donation in France).
Recruitment	Fresh blood samples were collected from healthy donors and obtained from French Blood Establishment (Hôpital Crozatier, Paris, France). The patients were randomly selected and we did not get any information concerning age or gender of the donors.
Ethics oversight	A contract ("convention") has been established between French Blood establishment (EFS) and Institut Curie, in conformity with national regulations and ethical guidelines. Written informed consent was obtained for each healthy donors.

Note that full information on the approval of the study protocol must also be provided in the manuscript.

Clinical data

Policy information about clinical studies

All manuscripts should comply with the ICMJE guidelines for publication of clinical research and a completed CONSORT checklist must be included with all submissions.

Clinical trial registration	*Provide the trial registration number from ClinicalTrials.gov or an equivalent agency.*

Study protocol	*Note where the full trial protocol can be accessed OR if not available, explain why.*
Data collection	*Describe the settings and locales of data collection, noting the time periods of recruitment and data collection.*
Outcomes	*Describe how you pre-defined primary and secondary outcome measures and how you assessed these measures.*

Flow Cytometry

Plots

Confirm that:

☒ The axis labels state the marker and fluorochrome used (e.g. CD4-FITC).

☒ The axis scales are clearly visible. Include numbers along axes only for bottom left plot of group (a 'group' is an analysis of identical markers).

☒ All plots are contour plots with outliers or pseudocolor plots.

☒ A numerical value for number of cells or percentage (with statistics) is provided.

Methodology

Sample preparation	For pDC sorting, a magnetic enrichment (Human Pan-DC Pre-Enrichment Kit StemCell Technologies) was performed on PBMCs before staining for 15 min at 4°C. . For naive and memory CD4+ T cells sorting, CD4+T lymphocytes were purified from PBMCs by immunomagnetic depletion with the human CD4+T cell Isolation KitII (MiltenyiBiotec), followed by staining for 15 min at 4°C.<br><br>Pure population of cells or PBMCs were stained for 15 minutes at 4°C with corresponding antibodies and isotypes.
Instrument	BD FACS Aria (BD Bioscience)<br>LSR Fortessa instrument (BD Bioscience)<br>LSR II instrument (BD Biosciences)<br>FACS Vantage instrument (BD Biosciences)
Software	GraphPad Prism<br>FACS DIVA for sorting<br>FlowJo10 for analyses
Cell population abundance	All analyzed cell populations had a purity over 95 %. Neutrophils abundance in PBMCs was around 30%. All other cell types mentionned in the current study were sorted with a purity over 95 % for the analyses.
Gating strategy	Neutrophils were identified among activated PBMCs as CD15+ cells.<br><br>Gating strategy for pDC sorting from PBMCs: After standard gating to eliminate debris, and doublets, we excluded lineage positive cells (containing CD3, CD14, CD16, and CD19 markers). Among the cells negative for lineage markers, we selected pDC as CD4+ CD11c- cells.<br><br>Gating strategy from memory and naive CD4+ T cells sorting from PBMCs : After standard gating to eliminate debris, doublets, and dead cells, we excluded CD25+ cells and CD4- cells. Among the CD25-CD4+ cells, we selected CD45RA+CD45RO- cells as naive CD4+ T cells, and CD45RA-CD45RO+ cells as memory CD4+ T cells.

☒ Tick this box to confirm that a figure exemplifying the gating strategy is provided in the Supplementary Information.
